# Understanding common human driving semantics for autonomous vehicles

**DOI:** 10.1016/j.patter.2023.100730

**Published:** 2023-04-18

**Authors:** Yingji Xia, Maosi Geng, Yong Chen, Sudan Sun, Chenlei Liao, Zheng Zhu, Zhihui Li, Washington Yotto Ochieng, Panagiotis Angeloudis, Mireille Elhajj, Lei Zhang, Zhenyu Zeng, Bing Zhang, Ziyou Gao, Xiqun (Michael) Chen

**Affiliations:** 1 Institute of Intelligent Transportation Systems, College of Civil Engineering and Architecture, Zhejiang University, Hangzhou 310058, China; 2 Polytechnic Institute & Institute of Intelligent Transportation Systems, Zhejiang University, Hangzhou 310015, China; 3 School of Medicine, Zhejiang University, Hangzhou 310058, China; 4 Alibaba-Zhejiang University Joint Research Institute of Frontier Technologies, Hangzhou 310027, China; 5 School of Transportation, Jilin University, Changchun 130022, China; 6 Department of Civil and Environmental Engineering, Imperial College London, South Kensington Campus, London SW7 2AZ, UK; 7 Alibaba Group, Hangzhou 310052, China; 8 School of Traffic and Transportation, Beijing Jiaotong University, Beijing 100044, China; 9 Zhejiang University/University of Illinois Urbana-Champaign (ZJU-UIUC) Institute, Zhejiang University, Haining 314400, China; 10 Zhejiang Provincial Engineering Research Center for Intelligent Transportation, Hangzhou 310058, China

**Keywords:** human-machine interaction, neuroscience, hierarchical understanding abstraction, electroencephalography, neural-informed model, driving behavior perception, driving semantics, autonomous vehicle

## Abstract

Autonomous vehicles will share roads with human-driven vehicles until the transition to fully autonomous transport systems is complete. The critical challenge of improving mutual understanding between both vehicle types cannot be addressed only by feeding extensive driving data into data-driven models but by enabling autonomous vehicles to understand and apply common driving behaviors analogous to human drivers. Therefore, we designed and conducted two electroencephalography experiments for comparing the cerebral activities of human linguistics and driving understanding. The results showed that driving activates hierarchical neural functions in the auditory cortex, which is analogous to abstraction in linguistic understanding. Subsequently, we proposed a neural-informed, semantics-driven framework to understand common human driving behavior in a brain-inspired manner. This study highlights the pathway of fusing neuroscience into complex human behavior understanding tasks and provides a computational neural model to understand human driving behaviors, which will enable autonomous vehicles to perceive and think like human drivers.

## Highlights

• Reveal human auditory cortex activation during driving
• Discover the hierarchical structure of human driving understanding
• Propose a neural-informed semantics-driven driving understanding model
• Address long-term contextual dependency of driving behaviors

## The bigger picture

“Driving like humans” is the ultimate goal of autonomous driving. Hence, human-like driving understanding ability is required for autonomous vehicles to better understand the driving behaviors of surrounding human-driven vehicles. In this study, we investigated human driving neural response and subsequently built a biologically plausible model to interpret driving behaviors like humans. This study pioneers the design of bio-inspired, human-like autonomous vehicles and can ultimately benefit future research of human-machine interactions.

## Teaser

Autonomous vehicles will share roads with human-driven vehicles and bring with them problems regarding bidirectional understanding of driving behavior. Based on cerebral neurological findings from the human process for understanding driving, a novel neural-inspired semantics-driven driving understanding model is proposed for autonomous vehicles. The model imitates the way humans understand driving and can interpret long-term driving behavior evolutions like human drivers.

## Introduction

Autonomous vehicles (AVs) continue to receive significant attention worldwide because they have the potential to realize a safer, faster, and more efficient mode of transportation. Every day, almost 2,700 people are killed globally in traffic crashes^6^; fatal and non-fatal crash injuries are estimated to cost approximately 1.8 trillion US dollars between 2015 and 2030.^7^ By shifting vehicle control from the human driver to machines via AVs, driver-related road crashes can be eliminated and thus save lives.^8^ However, until the transition to fully autonomous transport is completed, AVs will inevitably share roads with human-driven vehicles. During this transitory phase, AVs and human-driven vehicles need to share mutually interactive behaviors.^9^^,^^10^ Given this context, it is impossible to expect every human driver to accommodate certain traits and attributes of AVs, such as inconsistent or stilted driving behaviors (e.g., aggressive car following, jerking, sudden braking, or unexpected mandatory lane changing). Existing studies have revealed that a lack of transparency in AV decision making creates a psychological barrier that affects human drivers’ trust in AVs^11^; human drivers expect AVs to mimic their driving behaviors to become trustworthy.^12^ A more plausible approach is for AVs to acquire the ability to drive like human drivers, which would make it easier for other road users to interpret their driving behaviors and react appropriately. This would subsequently rebuild the driver’s trust and increase the social acceptability of AVs.^13^^,^^14^

**Figure 1:**
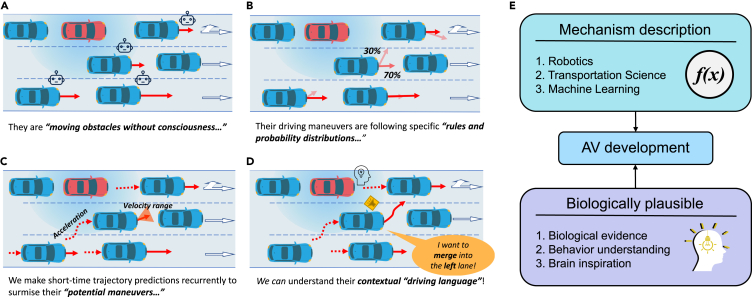
Four-stage development to understand the driving behavior of AVs (A) The surrounding vehicles are considered moving obstacles without self-consciousness. (B) The velocity of the surrounding vehicles is predicted using probability distribution outputs of discrete choice models. (C) The potential maneuvers of surrounding vehicles are surmised by recurrently applying short-time trajectory prediction models. (D) The intentions of the surrounding vehicles are understood from their contextual driving behaviors. (E) The mechanistic and biological requirements for AV development.

In recent years, various types of AVs were developed and tested in urban road scenarios, and they yielded promising results and applications.^15^^,^^16^^,^^17^ Given that vehicular sensing and navigation technologies are relatively mature,^18^ major concerns with AV adoption are related to whether AVs can interact appropriately with the human-driven vehicles in the surrounding areas. However, research studies on understanding common driving behaviors and designing AVs to operate while following human-like principles or brain-inspired mechanisms remain lacking. Therefore, we developed a method to understand AV driving behaviors as shown in Figure 1, where the red vehicle represents an AV and the blue vehicles represent the surrounding human-driven vehicles in a typical driving scenario.

Unlike the classical vehicular trajectory prediction and route planning models^19^^,^^20^^,^^21^ widely accepted in the robotics field (Figure 1A) or the various discrete-choice driving models^22^^,^^23^ developed in the traffic engineering discipline (Figure 1B), recent research^24^^,^^25^^,^^26^ showed that subjective individual human driving factors are critical and cannot be neglected in the development of human-like AVs (Figure 1C). Unfortunately, as human driving behaviors evolve with an indefinite temporal dependency, state-of-the-art machine learning-based methods that partially imitate the nature of the human driving decision-making process may lose the ability to adapt and generalize. For example, standalone data-driven intention recognizers (e.g., deep neural networks) employed in machine learning-based methods are likely to be trapped in the following dilemma: those with increased temporal inputs carry a more significant risk of overfitting local features and the output confusing driving intentions, whereas those with shorter temporal dependencies are too myopic to fully understand the driving intentions of surrounding drivers, such as human drivers.

Understanding common human driving behaviors that follow the human cerebral driving thinking mechanisms of human drivers (Figure 1D) is necessary to address this dilemma.^27^^,^^28^^,^^29^ As human driving behaviors are generated by humans rather than machines, AV development needs to be mechanistically and biologically plausible (Figure 1E).

Our research is motivated by the fact that talking while driving can cause severe distractions because both behaviors or activities share the same cerebral resources^30^^,^^31^ (right parietal resources^32^ and working memory in the prefrontal cortex^33^^,^^34^). Therefore, we attempt to fuse neuroscience and robotics in a neuroengineering manner^35^ in this study. To this end, we designed two separate electroencephalography (EEG) experiments to reveal the formation of cerebral driving thinking and evolution using well-studied linguistic analyses. We subsequently present a semantics-driven method to understand common driving behaviors for developing AVs in a brain-inspired manner. First, we compared the cerebral activities involved in human linguistic and driving thinking with EEG and reported biological evidence that they share conspecific neural activations in the auditory cortex. Second, we designed another EEG experiment by watching sequential and randomized driving videos at a 4 Hz frame rate by following similar research pipelines in a previous study^36^ and verifying whether driving intentions can be hierarchically and semantically understood. Proposed comparisons from intention decoding to action formation revealed similar cortical activations and presented a sound research direction for semantically addressing human driving thinking. This idea was also supported by several cerebral and cognitive studies,^37^^,^^38^^,^^39^ which indicated that understanding common driving behavior using natural language processing (NLP) techniques, especially semantics-driven methods, was biologically plausible. We prototyped the approach to demonstrate that the proposed semantics-driven method for understanding common human driving behaviors for AVs in a linguistic-inspired manner is mechanistic and biologically plausible.

Neurological and cognitive research^40^ has revealed that the cognitive mechanism of humans requires both the perceptual encoding of the stimulus and its subsequent maintenance in the working memory for further processing. Inspired by the human cerebral function to process and understand speech,^41^ the proposed prototypical implementation method imitates the cerebral pathway, which a human driver uses to understand the contextual “driving language” of surrounding vehicles. This helps us to gain insights into employing grammatical or linguistic knowledge for addressing the nature of human driving thinking. First, detailed driving behaviors (namely, common driving syllables) are abstractly represented by meaningful common driving lexical units and phrasal units using a brain-inspired codebook by imitating the cortical encoding manner of human speech units.^42^ Second, these phrasal units further form common driving sentences based on the behavioral chain of each vehicle to deal with long-term contextual dependency. Third, the sentences are compared and analyzed at the document level using the latent Dirichlet allocation (LDA) model^43^ to find language stimuli^44^^,^^45^; they are then projected into common driving topics in a high-dimensional feature space. Finally, a biologically plausible spiking neural network (SNN)^46^ is used to understand contextual common driving semantics, such as those of human drivers, by utilizing the abstracted evolution characteristics of the common driving topics in the projected feature space. Our experimental results provide computationally explicit evidence that the proposed demonstration can accurately capture and understand common human driving topic evolution in a flexible time and that it can understand and predict potential stop-and-go or lane-changing behaviors semantically in a human-like manner.

Our work includes the following key features.

(1) We present biological findings that human cerebral driving understanding activates similar hierarchical linguistic neural functions in the auditory cortex as that with linguistic understanding.
(2) A semantics-driven framework is proposed to understand common driving behaviors in a human-like and linguistic-inspired manner contextually. This provides AVs with the ability to perceive and think similar to human drivers.
(3) A prototype is proposed to demonstrate the possible computational neural implementation of the biological findings. The evolution of human driving behavior is encoded to highly abstracted common driving units and topics in a brain-inspired manner, and it is understood using an SNN with NLP techniques to tackle the contextual reasoning limitations of state-of-the-art human-like driving behavior understanding methods and models.

## Results

### EEG comparisons of driving and linguistic understanding

**Figure 2:**
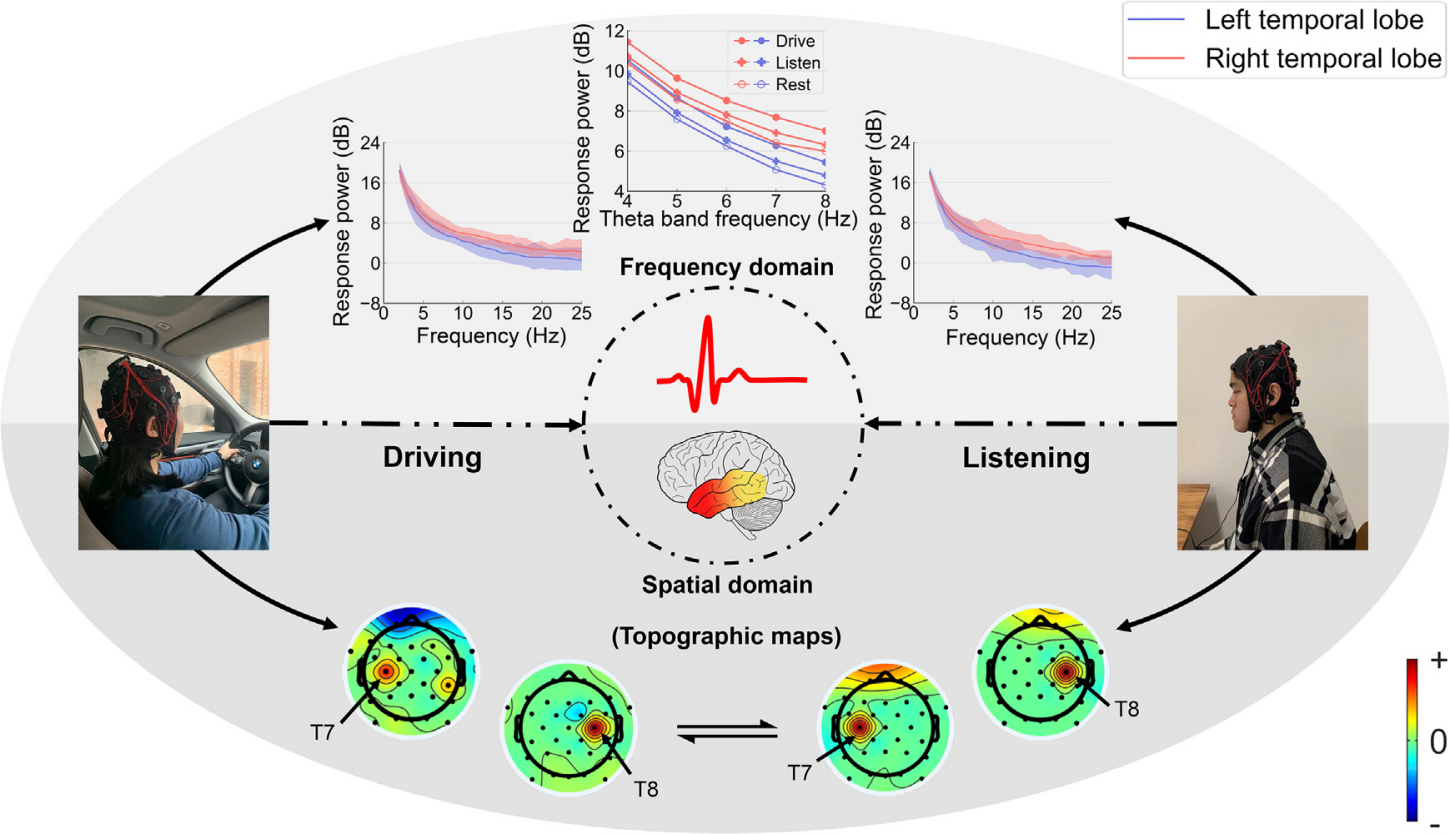
EEG comparisons between driving and listening activities The bottom half shows part of the topographic maps of the driving and listening tasks obtained using independent component correlation algorithm (ICA). Both driving and listening activities cause spatial activation of the T7 and T8 in the temporal lobes (auditory cortex). The top half shows the corresponding power comparison in the frequency domain. The two lower line charts represent the power intensities of the temporal lobes from 2 to 25 Hz. The lines indicate the average values, and shaded areas indicate the corresponding value ranges. The middle line chart represents the power comparison of listening, driving, and resting with respect to the frequency in the theta band (4–8 Hz) in each temporal lobe.

We collected EEG responses from 18 participants on three 5-min tasks—resting, driving, and listening—to investigate the relationship between human linguistics and driving-related thought processes. We compared the collected responses in the spatial and frequency domains. Counterintuitively, as reflected in the topographic maps (bottom half of Figure 2), the spatial domain analysis shows that driving in a silent environment activates both temporal lobes, similar to that for a listening task. In addition, we plotted the cortical response power spectrum for each temporal lobe separately, as shown in the top half of Figure 2. The visual analysis showed that both activities had analogous power intensities and trends. We further examined theta band responses for the driving, listening, and resting tasks and plotted their mean value with respect to frequency in the middle line chart of the top half of Figure 2 because the phase patterns of the theta band (4–8 Hz) responses in the right human auditory cortex are closely related to the inherent brain rhythms of speech comprehension.^47^ The results suggest that the driving task has significantly higher neural responses in each temporal lobe in the theta band (4–8 Hz) compared with that in the resting state (p < 0.05, paired one-sided t test with false discovery rate [FDR] correction for multiple comparisons; see Table S1 for details). This biological finding suggests that human driving understanding probably activates neural functions in the auditory cortex, which are widely believed to occur during linguistic understanding tasks.

### Hierarchical structure of driving understanding

**Figure 3:**
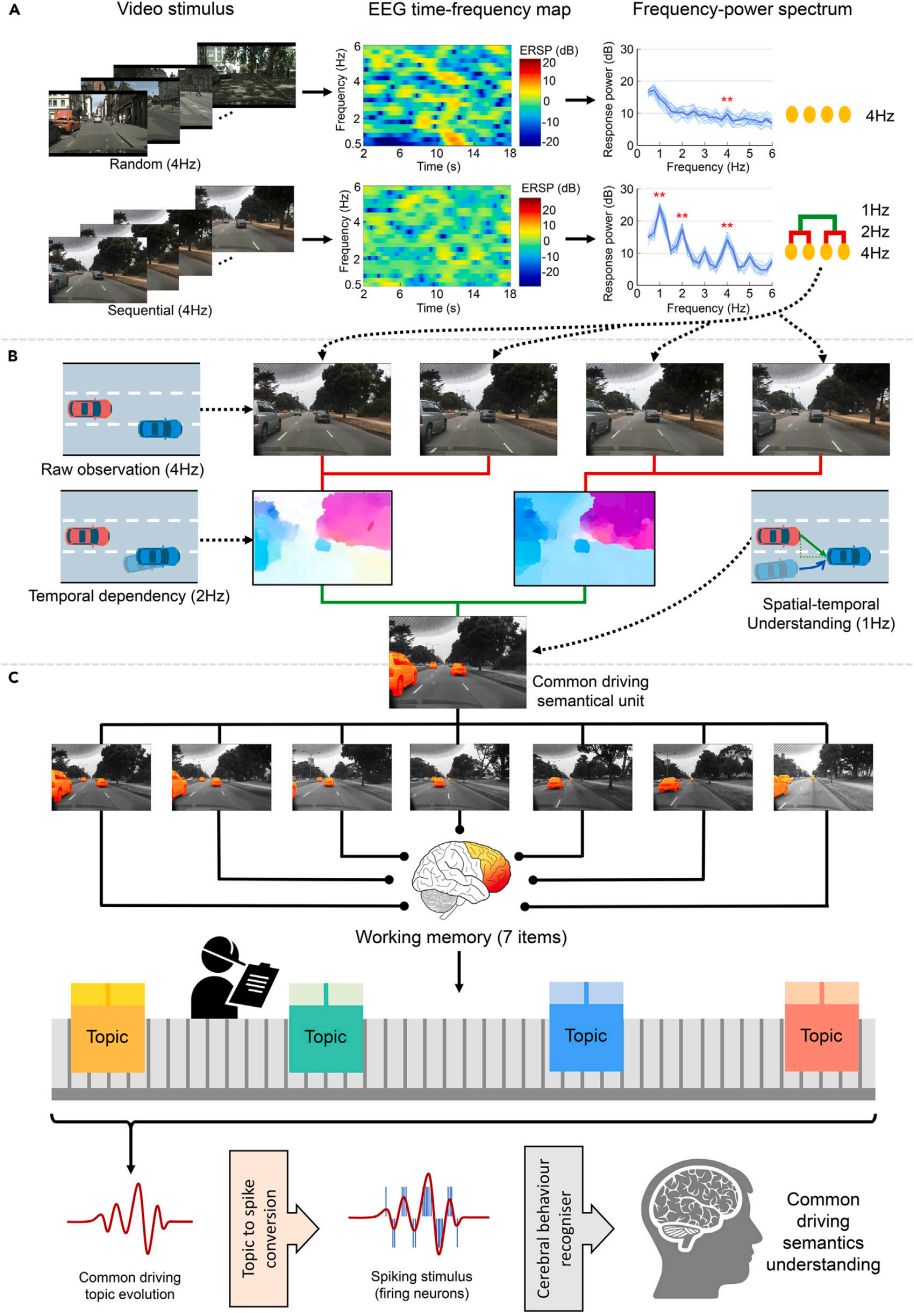
Brain-inspired, semantics-driven framework for human driving understanding (A) Biological evidence of tracking hierarchical structures in human cerebral driving understanding. EEG time-frequency maps, frequency-power spectra, and hierarchical structure representations corresponding to different video stimuli are shown. The light curves in the frequency-power spectrum represent individual responses, and the dark curve represents the grand average. Frequency bins with significantly stronger power than neighbors (0.5 Hz range) are marked (∗∗p < 0.01). (B) The three-level representation scheme of human driving understanding for forming common driving semantical units. Two-level abstractions are marked with red and green lines, respectively. (C) Semantics-driven driving understanding scheme. The sequential common driving semantical units are used to form common driving topics in the working memory, and the driving semantics are recognized by a cerebral behavior recognizer.

The most critical attribute of human linguistic understanding is its combinatorial nature: the grammatical system relies on hierarchical linguistic representations (e.g. words, phrases, and sentences) to understand intrinsic semantical knowledge.^36^ Therefore, we investigated the hierarchical structure of driving understanding and designed a brain-inspired, semantics-driven understanding framework for driving thinking, as shown in Figure 3. First, we synthesized three isochronous, 4-Hz first-person driving video clips—two with sequential frames and the others with random frames (Videos S1, S2, and S3). The EEG responses of the participants when watching these low-frame-rate video clips were recorded separately. A typical corresponding EEG time-frequency map and frequency-power spectrum are shown in Figure 3A. For the stimuli involving sequentially driven video frames, we observed significant rhythmic patterns in event-related spectral perturbation (ERSP) and distinctive spectral peaks at 2 and 1 Hz (stronger power than neighbors, 0.5-Hz range), respectively (p < 0.05, paired one-sided t test with FDR correction for multiple comparisons; see Table S2 for details). This suggests that the driving understanding task activates neural responses at different hierarchical levels. Hence, we conclude that the phenomenon is consistent with the hierarchical structure of listening to speech reported by Ding et al*.*^36^

Video S1. Synthesized 4-Hz driving video clips with sequential frames

Video S2. Synthesized 4-Hz driving video clips with sequential frames

Video S3. Synthesized 4-Hz driving video clips with random frames

This finding indicates that human drivers can employ similar intrinsic hierarchical linguistic structures (i.e., word, phrase, and sentence levels) in the cortex to pre-process an abstract dynamic driving scenario instead of directly utilizing raw driving information as part of the decision-making unit. In a correlative experiment, the neural responses of random driving video frames were relatively smooth, which eliminated the phrasal/sentential structure. The corresponding frequency-power visualization suggested that participants merely obtained any abstracted knowledge from these irrelevant frames; this strengthened the finding of hierarchical driving understanding abstraction in the human brain.

### Semantics-driven framework to understand human driving thinking

Inspired by the hierarchical structure of driving behavior understanding, we proposed a three-level common driving semantics structure (corresponding to syllabic, lexical, and phrasal levels in linguistic structure) to encode raw driving information into meaningful and comprehensible semantic units, as shown in Figure 3B. After obtaining the raw environment observations during driving, we used the information from two adjacent observations to infer the temporal dependency of the preceding vehicles. Then, we used sequential temporal dependencies to acquire the spatial-temporal understanding of the driving environment. The proposed encoding method works in a pipeline similar to how the cortex ensembles neurons to encode semantic knowledge.^48^ This bridges the raw information processing of human linguistic understanding and the AV’s driving behavior understanding.

Encoded common driving semantic units are temporally stored in the working memory and then used to understand common driving semantics, as shown in Figure 3C. Then, these sequential common driving semantic units in the episodic buffer are processed to retrieve common driving topics in a recurrent manner. The retrieved common driving topic time series reflects the contextual evolution of the drivers’ understanding of driving. To make this evolution understandable and interpretable, we converted the common driving topics into neural spikes (i.e., a set of firing neurons in the cortex) and introduced cerebral behavioral recognizers to fully imitate the understanding and decision-making functions of biological brains.^49^ Thus, human cerebral driving thinking is partially revealed and imitated, which can provide a biologically plausible, semantics-driven framework and facilitate the design of next-generation human-like AVs.

### Understanding the evolution of common driving semantics

**Figure 4:**
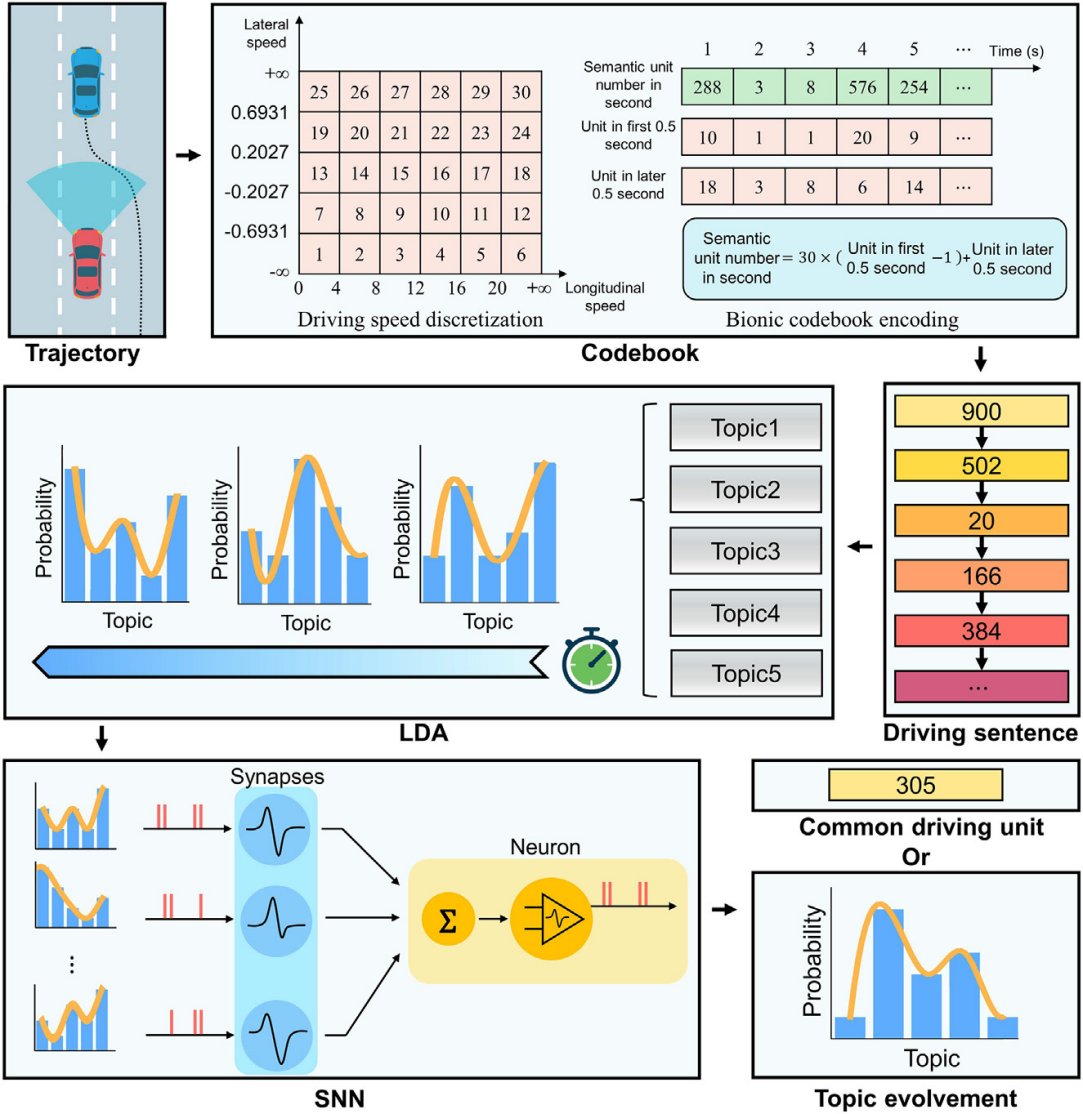
Prototypical implementation method for understanding common driving semantics The trajectory of the surrounding vehicle is encoded by a bio-inspired hierarchical codebook and formed into driving sentences. LDA and SNN then process the driving sentence to obtain either the upcoming common driving unit or the topic evolution.

To validate whether the proposed semantics-driven framework can effectively understand human driving evolution, we designed a prototypical implementation experiment using a semantics-driven approach to understand human driving intentions with ubiquitous traffic eye (UTE) naturalistic driving trajectory datasets^4^^,^^5^ (see experimental procedures for details). We used the Shuangqiaomen Expressway, Nanjing, China (SQM1) dataset in this experiment, which contains both free-flow and congested-flow traffic with a high proportion of lane-changing vehicles (see Figure S1 for the site layout and trajectory map). A pipeline of the proposed prototypical implementation method is shown in Figure 4. As human drivers perceive the driving environment visually with discrete fuzzy thresholds rather than precise measurements,^50^^,^^51^ we proposed a discrete, brain-inspired codebook to form common driving semantic units that imitate the hierarchical abstraction process and acquire behavioral semantics.^52^^,^^53^ After encoding each vehicle trajectory into discrete common driving semantic units, we translated the vehicular trajectory into a driving document per vehicle. The driving document was used to retrieve a common driving topic time series using an LDA model recurrently in an unsupervised manner (see Note S1, Figure S2, and Table S3 for details). The buffer length of the working memory was selected as seven based on previous biological evidence.^54^ Then, we employed an SNN as the cerebral behavior recognizer and trained the SNN model to predict topic evolution recurrently in a self-supervised and natural language generation manner^55^^,^^56^ (see Note S2, Figure S3, and Table S4 for details).

**Figure 5:**
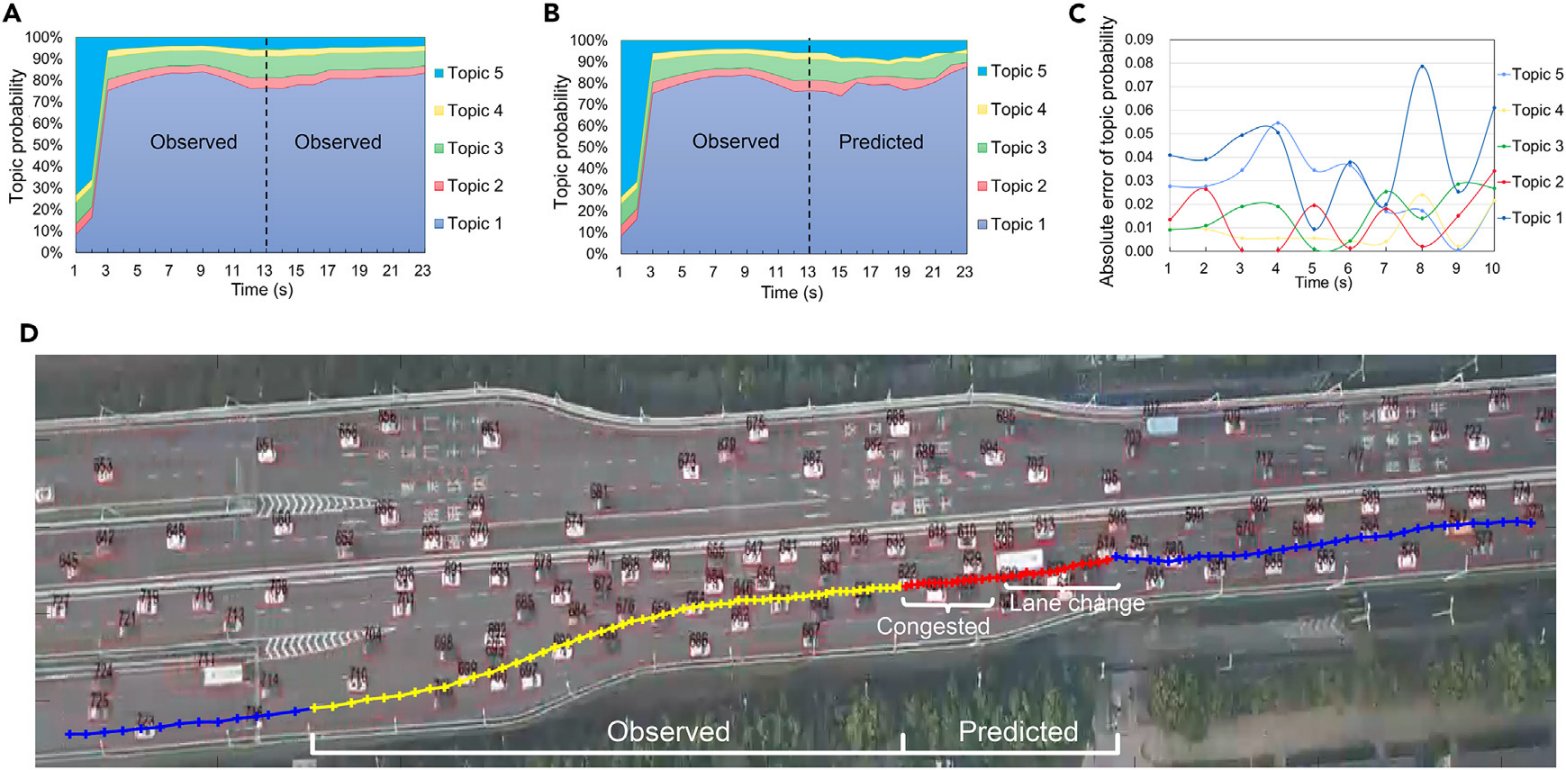
Visualization of multi-step driving topic evolution prediction (A) Observed topic evolution (1–23 s) for target vehicle #573 in the SQM1 dataset. (B) Predicted topic evolution (13–23 s) of the target vehicle. (C) Absolute difference between predicted and observed topic probability values. (D) Visualization of the corresponding vehicle trajectory. The yellow dotted line is the observed trajectory, and the red dotted line is the predicted trajectory containing congested and lane-changing driving behaviors. Each dot denotes a period corresponding to six video frames (0.33 s each).

Our experimental results indicate that the proposed method achieved high accuracy in recurrently predicting the most relevant topic (1^st^ second: 98.14%, 5^th^ second: 95.33%, and 10^th^ second: 95%) while retaining a low root-mean-square error (RMSE; 1^st^ second: 0.06, 5^th^ second: 0.09, and 10^th^ second: 0.10; see Table S5 for details). A visual assessment of Figure 5 suggests that the proposed method captured stop-and-go driving behaviors and predicted lane-changing maneuvers in the next few seconds (topic group during the 17^th^ through the 21^st^ second) without explicitly pre-defining the potential lane-changing choice probability. This indicates the advantage of the long-term contextual prediction stability of the proposed semantics-driven understanding framework.

### Semantics-driven multi-step vehicle velocity prediction

**Figure 6:**
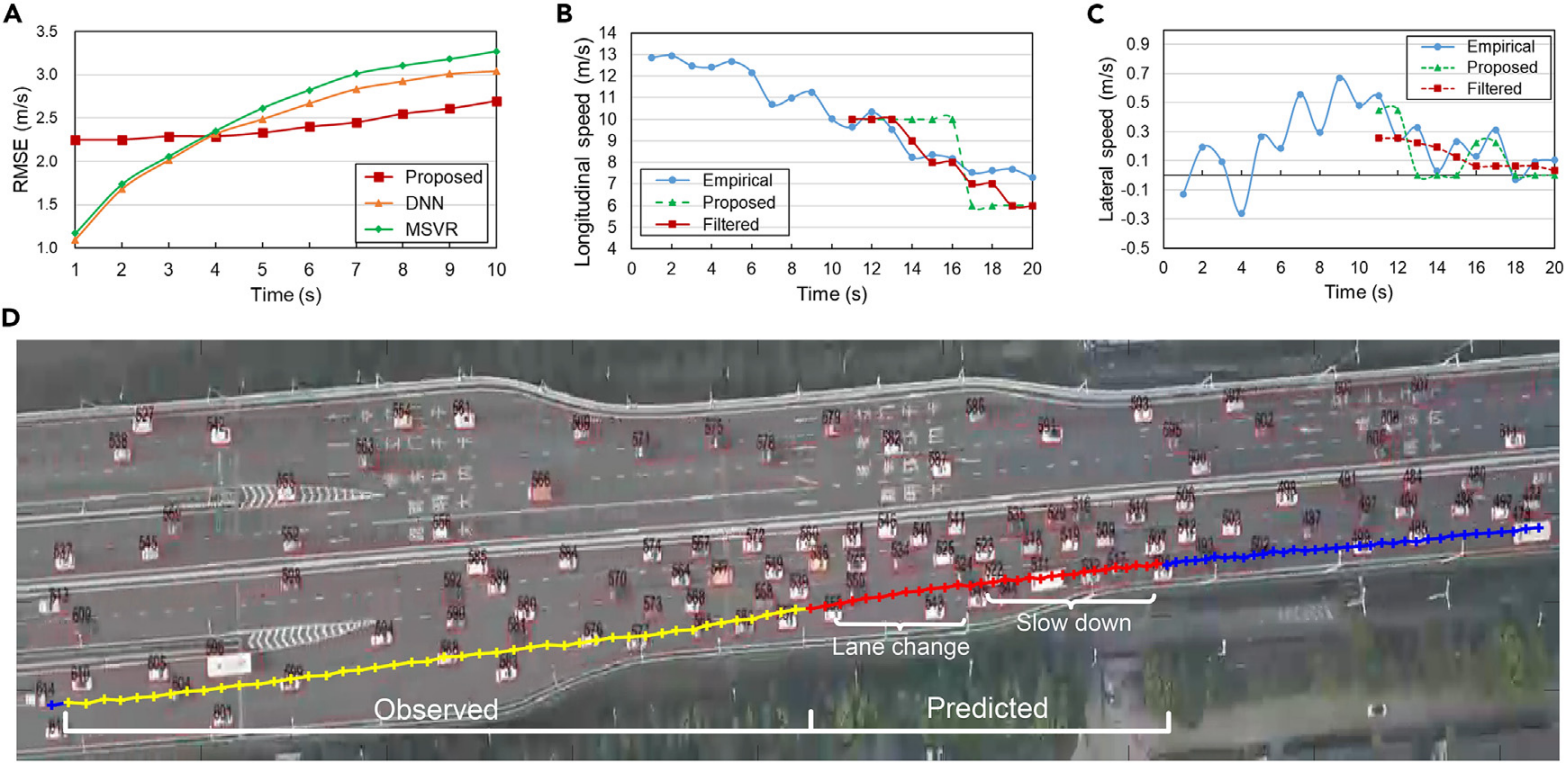
Visualization of multi-step vehicle velocity prediction (A) Overall accumulated RMSE value of the proposed method, DNN, and MSVR in multi-step prediction. (B) Observed, predicted, and filtered longitudinal speeds of vehicle #478 in the SQM1 dataset. (C) Observed, predicted, and filtered lateral speeds of the vehicle. The positive values refer to moving left and negative values to moving right. (D) Visualization of corresponding vehicle trajectory. The yellow dotted line is the observed trajectory, and the red dotted line is the predicted trajectory containing lane-changing and deceleration driving behaviors. Each dot denotes the duration of six video frames (0.33 s each).

We used similar training pipelines to validate the effectiveness of the multi-step velocity prediction using the proposed method. We used the mean speed in each semantic unit to calculate the RMSE of the speed-prediction task. We compared our experimental results with a deep neural network (DNN) and multi-output support vector regression (MSVR) in Figure 6A and using the RMSE metric (see Tables S6 and S7 for details). The RMSE of the proposed method was relatively high because the discrete nature of the common driving unit encoding process may induce measurement errors. However, as we predicted the vehicle velocity recurrently, the RMSE of the comparative models accumulated rapidly and surpassed the proposed method in the 4^th^ second. In contrast, the RMSE of the proposed method remained consistent. This comparison explicitly confirms that the proposed framework has excellent robustness in predicting contextual driving behaviors in a flexible time and has outstanding generalization ability, which helps overcome the dilemma faced by standalone data-driven intention recognizers.

To further validate the velocity prediction accuracy, we visualized the velocity prediction result of a typical vehicle in Figures 6B–6D. Although the proposed model generates multi-step discrete speed prediction according to the mean value of each bin in the brain-inspired codebook, the vehicle cannot adjust its longitudinal or lateral speed instantaneously as the step function indicates. We employed a uniform filter to smooth the outputs and demonstrate the practical speed changes in both directions. A visual assessment of the results showed that filtered speed in both directions was close to that of the observed trajectory and could precisely reflect lane-changing and deceleration maneuvers. Therefore, we concluded from the common driving topic evolution understanding and vehicle velocity prediction results that the proposed semantics-driven understanding framework for human driving thought process was biologically and computationally sound.

## Discussion

This article addressed the future coexistence challenge of autonomous and human-driven vehicles and proposed a brain-inspired computational neural framework to understand the evolution of common driving topics and address this challenge. We reported biological findings that driving and linguistic understanding share conspecific hierarchical neural abstractions in the auditory cortex and further proposed a semantics-driven method to understand human driving thinking. The prototypical implementation experimental results showed that the proposed framework accurately predicted driving maneuvers in the next few seconds while retaining a lower error accumulation rate benefiting from contextual driving semantics compared with other data-driven trajectory prediction baselines. Therefore, this work acts as a complementary component for current AVs, and it can provide AVs with the ability to perceive and think similar to human drivers. We lay the biological and theoretical foundations to advance the development of human-like AVs.

The goal of AVs is to drive similar to human drivers. AVs and the surrounding human-driven vehicles should have a clear bidirectional understanding of each other’s driving intentions. State-of-the-art driving intention recognition models have endlessly improved the accuracy of trajectory prediction using highly stacked and complex neural networks. However, their model interpretability decreases quickly, which can create barriers to mutual human-machine understanding and result in traffic conflicts or accidents. Our work pioneers the introduction of biological and cerebral research methods for analyzing human driving understanding patterns related to driving behaviors and provides a prototypical demonstration to benefit future human-machine interaction research.

There are some limitations in our study that should be addressed in future research. For example, the proposed method focuses on developing frameworks that would allow AVs to comprehend and interpret the driving behaviors of surrounding vehicles, similar to human drivers. However, the method does not address the static driving environment quantification problem. Weather, traffic signs, lane specifications, etc., strongly correlate with human driving behavior adaptation. Thus, an important future direction would be to quantify cerebral responses to various driving environment factors in a stimulus-based manner and to add them to the common driving semantics. Moreover, our work represents the first step toward developing brain-inspired, human-like AVs and addressing vehicular interactions. Therefore, future work should include a self-correcting mechanism that can actively consider the effect of contextual vehicular interactions in an attention-based manner.

Autoencoders (AEs) that use machine learning algorithms, such as variational AEs, sparse AEs, and semantic AEs, can be embedded into the common semantic unit encoding process to achieve a higher-level abstracted semantic representation. Theoretically, these AEs are more biologically sound to imitate the activation of cerebral neurons and fit continuous velocity changes compared with that using the proposed discrete codebook. The employment of AEs neither improves method interpretability nor decreases computational complexity. Therefore, in this study, AEs were replaced by a more straightforward, yet effective, codebook method following the model simplicity law of Occam’s razor.^57^

## Experimental procedures

### Resource availability

#### Lead contact

Further information and requests for resources and reagents should be directed to and will be fulfilled by the lead contact, Xiqun (Michael) Chen (chenxiqun@zju.edu.cn).

#### Materials availability

This study did not generate new unique materials.

### Ethical approval

The experimental procedures were approved by the Research Ethics Committee of the School of Medicine of Zhejiang University (ZGL202204-7). This study was conducted in accordance with the ethical standards of the 1964 Declaration of Helsinki. All participants provided written informed consent before the experiment, and the possible consequences of the study were explained.

### Participants

Eighteen normal-hearing, right-handed adult drivers (20–36 years old, mean 24.72 years old; 8 female) participated in the EEG experiments. All participants were qualified drivers with driving licenses, and their average driving experience was 4.5 years. The sample size for previous linguistic experiments was between 3 and12,^36^^,^^41^ and the basic phenomenon reported here was replicated in all EEG experiments in this study.

### EEG data processing

The EEG signals were recorded at 128 samples per second (1,024-Hz internal) with a 32-channel Emotiv Epoc Flex device and processed with EEGLAB software.^58^ The electrodes were positioned at Cz, Fz, Pz, Oz, FP1, FP2, F3, F4, F7, F8, FC1, FC2, FC5, FC6, C3, C4, FT9, FT10, T7, T8, CP5, CP1, CP2, CP6, P3, P4, P7, P8, PO9, PO10, O1, and O2, based on the standard 10–20 international EEG system (also known as IS). The raw signals were filtered by a band-pass finite impulse response (FIR) filter (0.5–50 Hz) to remove noise and segmented in 2-s epochs for analysis. Second, interpolation was performed with adjacent electrodes, and the data were rereferenced by computing the average reference. Finally, the signals were decomposed using an independent component correlation algorithm (ICA),^59^ and artifacts such as electrooculograms (EOGs) were removed.

### EEG experiment 1

Each participant was asked to listen to the news materials: “News 1 + 1: How to disinfect correctly in work and life?” (Mandarin)^60^ at a comfortable loudness level for 5 min. They then rested for 3 min, and the resting-state EEG was recorded for 5 min. Finally, they rested for another 3 min and drove the provided vehicle on a silent, closed road section for another 5 min. The EGG signals were recorded simultaneously. The comparison of EEG responses between the driving and resting tasks in the theta band (4–8 Hz) in each temporal lobe was analyzed using a paired one-sided t test (FDR corrected). The details are shown in Table S6.

### EEG experiment 2

We synthesized three video clips using the Cityscapes driving video dataset^2^^,^^3^ (Video S1. Synthesized 4-Hz driving video clips with sequential frames, Video S2. Synthesized 4-Hz driving video clips with sequential frames, Video S3. Synthesized 4-Hz driving video clips with random frames). The frame rate was 4 Hz for all video clips, and the frames were arranged in either a sequential (two video clips) or random (one video clip) order. The video clips were played on a desktop computer screen without sound. Each participant was instructed to watch the video clips randomly with two 3-min breaks. The EGG signals were recorded simultaneously. The significance of distinctive EEG spectral peaks (at 4, 2, and 1 Hz) was examined by testing if the neural response power in the target bin was significantly stronger than the average of their corresponding neighboring 4 frequency bins (two bins on each side, 0.5-Hz range) using paired one-sided t test (FDR corrected). The details of this power analysis are shown in Table S7.

### Driving trajectory dataset

UTE naturalistic driving trajectory datasets^4^^,^^5^ were used in the experiments to evaluate the proposed semantics-driven model quantitatively. We used the vehicle trajectories captured from the SQM1 dataset for model evaluation. The SQM1 dataset was captured by an unmanned aerial vehicle (UAV) at an altitude of 310 m using aerial photography. It consisted of 822,712 trajectory points from 1,041 vehicles collected from a road section of 427 m. These data provide precise vehicle position coordinates with a time resolution of 0.1 s and contain the speed, acceleration, spacing, time distance, lane, etc.

### Brain-inspired codebook

The two-dimensional vehicle velocity (i.e., longitudinal and lateral speeds) was sampled every 0.25 s (4 Hz) and averaged every 0.5 s in a pairwise manner (2 Hz). Using a discrete codebook, the 2-Hz averaged velocity was divided into 30 non-overlapping cells with different thresholds. The bin interval sets of the lateral and longitudinal speeds used in this study are shown in Figure 3. The evenly spaced lateral speed interval is mapped non-linearly by $v^{\prime }=\tanh\left(2v\right)$ (*v* stands for the uniform speed threshold, and *v'* denotes the mapped threshold) to balance the data distribution because vehicles tend to maintain their lane most of the time. After obtaining the bin numbers in the first and later 0.5 s, another codebook was constructed to represent the 1-Hz common driving semantic units with 900 bin numbers, which imitates the three-level abstraction process of human driving understanding.

## Data Availability

The processed EEG data used to generate the results and the prototypical implementation code to understand common human driving semantics of this study are publicly available at Zenodo (https://doi.org/10.5281/zenodo.7714338).^1^ The Cityscapes driving video dataset is publicly available on its website.^2^^,^^3^ The naturalistic vehicle trajectory dataset is publicly available from the UTE Project by Southeast University and can be either downloaded from its website^4^ or requested from the authors.^5^

## Supplemental information

Document S1. Notes S1 and S2, Figures S1–S3, and Tables S1–S7

Document S2. Article plus supplemental information
