# Supplementary material for: Understanding common human driving semantics for autonomous vehicles
Source: Patterns (N Y). 2023 Apr 18;4(7):100730. doi: 10.1016/j.patter.2023.100730 (PMC10382946; doi:10.1016/j.patter.2023.100730)
Supplement: Document S1. Notes S1 and S2, Figures S1–S3, and Tables S1–S7 [file mmc1.pdf]

Patterns, Volume *4*

Supplemental information

Understanding common human driving semantics for autonomous vehicles

Yingji Xia, Maosi Geng, Yong Chen, Sudan Sun, Chenlei Liao, Zheng Zhu, Zhihui Li, Washington Yotto Ochieng, Panagiotis Angeloudis, Mireille Elhajj, Lei Zhang, Zhenyu Zeng, Bing Zhang, Ziyou Gao, and Xiqun (Michael) Chen

Supplemental Information

Supplementary Notes

Note S1. Latent Dirichlet allocation model

The latent Dirichlet allocation (LDA) model was used in this study for common driving topic modeling purposes. As an unsupervised generative probabilistic model, LDA can adaptively analyze the structural features of the "document-word" relationship and output the document's topic distribution without any prior annotation information about the document. The joint distribution of all variables in the LDA is represented as follows:

$$P(w_i, z_i, \theta_i, \Phi|\alpha, \beta) = \prod_j^{V_j} P\left(w_{ij}\middle|\phi_{z_{ij}}\right) \cdot P\left(z_{ij}\middle|\theta_i\right) \cdot P(\theta_i|\alpha) \cdot P(\Phi|\beta), \quad (1)$$

where $P(\theta_i|\alpha)$ denotes the probability of generating topic distribution θ_i of document d_i by sampling from the Dirichlet prior distribution $D_K(\alpha)$ containing K topics, where α is the parameter of $D_K(\alpha)$; $P(z_{ij}|\theta_i)$ denotes the topic probability corresponding to the j-th word of document d_i generated by sampling from the multinomial distribution $M_K(\theta_i)$; $P(\Phi|\beta)$ denotes a "topic-word" distribution matrix in the Dirichlet distribution $D_{V_i}(\beta)$ containing V_i words for generating the topic z_{ij}, wherein β is the parameter of $D_{V_i}(\beta)$; $P(w_{ij}|\phi_{z_{ij}})$ denotes the probability of generating word w_{ij} by sampling from the multinomial distribution $M_V(\phi_{z_{ij}})$. The detailed document generation process is listed as follows:

Step 1: The topic distribution θ_i of document d_i is generated by sampling from Dirichlet distribution $D_K(\alpha)$ with K topics. Furthermore, the topic $z_{ij} \in [1,\ K]$ of the j-th word of document d_i is generated by sampling from the multinomial distribution $M_K(\theta_i)$.

Step 2: The word distribution $\varphi_{z_{ij}}$ of topic z_{ij} is generated by sampling from Dirichlet distribution $D_{V_i}(\beta)$ with V_i words and parameter β. Further, word w_{ij} is generated by sampling from multinomial distribution $M_V(\varphi_{z_{ij}})$.

The "document-topic" and "topic-word" distribution parameters can be optimized by performing LDA inference based on the online variational Bayesian algorithm. In this study, the best number of topics is tuned to be eight based on the perplexity metric (see Figure S2). The parameter settings for the model are provided in Table S3.

Note S2. Spiking Neural Network

Based on the abstracted common driving topic, a spiking neural network (SNN) was used to continuously predict the evolution trend of driving topics and the changes of driving speed at different timeframes in the future. This study constructed two SNNs based on four fully connected layers and a layer of leaky integrate-and-fire neurons (see Figure S3) to predict driving topic evolvement and vehicle velocity. The network construction of the two SNNs was identical except for the model's output. In an SNN, the input was first mapped and transformed through the fully connected layer $fully(\cdot)$:

$$X_{t+1} = fully(X_t) = W_t X_t, \tag{2}$$

where X_t denotes the input of the t-th fully connected layer of SNN, and X_0 represents the original feature input (i.e., common driving topics). The final layer of SNN consists of spiking neurons that perform the charging, discharging, and resetting operations via the following discrete equations:

$$H(t) = f\big(V(t-1),\ H(t-1)\big), \tag{3}$$

$$S(t) = \Theta(H(t) - V_{threshold}), \tag{4}$$

$$V(t) = H(t) \cdot \big(1 - S(t)\big) + V_{reset} \cdot S(t), \tag{5}$$

where $H(t-1)$ is the input received by the spiking neuron at time t, $H(t)$ and $V(t)$ denote the latent state of neurons and the membrane potential at time t, respectively. $V_{threshold}$ denotes the trigger threshold of the potential, and V_{reset} denotes the reset potential. $f(\cdot)$ denotes the charging equation of the spiking neuron, and the charging equation corresponding to the Leaky Integrate-and-Fire (LIF) neuron used in this study is as follows:

$$H(t) = V(t-1) + \frac{1}{\tau}(H(t-1) - V(t-1) - V_{reset}), \tag{6}$$

where τ denotes the membrane time constant, $S(t)$ is a spike fired by a neuron at time t. $\Theta(\cdot)$ denotes the spiking function, which is defined as follows:

$$\Theta(x) = \begin{cases} 1, & x \geq 0 \\ 0, & x < 0 \end{cases}. \tag{7}$$

If the $\Theta(x)$ value is one, a spike is fired, and the membrane potential is reset as V_{reset}. Otherwise, no spike is fired, and the membrane potential remains unchanged.

For the training process, we divide the training set, validation set, and test set to train the SNN with a ratio of 8:1:1, and use the 10-second historical driving topics as the input of the SNN to predict future driving topics and semantical driving units in the next ten seconds. The Adam optimizer is used to minimize the loss function of the SNN, and the mean square error (MSE) loss is used in this study, which can be defined as follows (we also provide the Mean Absolute Error (MAE) and Root Mean Squared Error (RMSE) that are used as performance measures in this study):

$$MSE = \frac{\sum_{t=1}^{N}(y_t - \hat{y}_t)^2}{N}, \tag{8}$$

$$MAE = \frac{\sum_{t=1}^{N}|y_t - \hat{y}_t|^2}{N}, \tag{9}$$

$$RMSE = \sqrt{\frac{\sum_{t=1}^{N}(y_t - \hat{y}_t)^2}{N}}, \tag{10}$$

where N denotes the total number of the test samples, y_t and $\hat{y}_t$ denote the observed and predicted values at time t, respectively. Detailed parameter settings for the SNN are presented in Table S4.

To verify and compare the performance of the proposed method in driving speed prediction, two classical models were used for experimental comparison, including deep neural network (DNN) and multi-output support vector regression (MSVR). Among them, DNN adopted the same network structure as SNN, and the difference is that it did not use the normalization and embed spiking neurons layer, and it employed raw velocity values as input and output instead of the proposed common driving topics. In addition, to make the original SVR suitable for solving the multi-step prediction task, we used a multi-output regression layer to encapsulate the basic SVR. We used the linear kernel function for feature mapping and extraction purposes in MSVR. The prediction and comparison results of each model are provided in Tables S5-S7.

Supplementary Figures

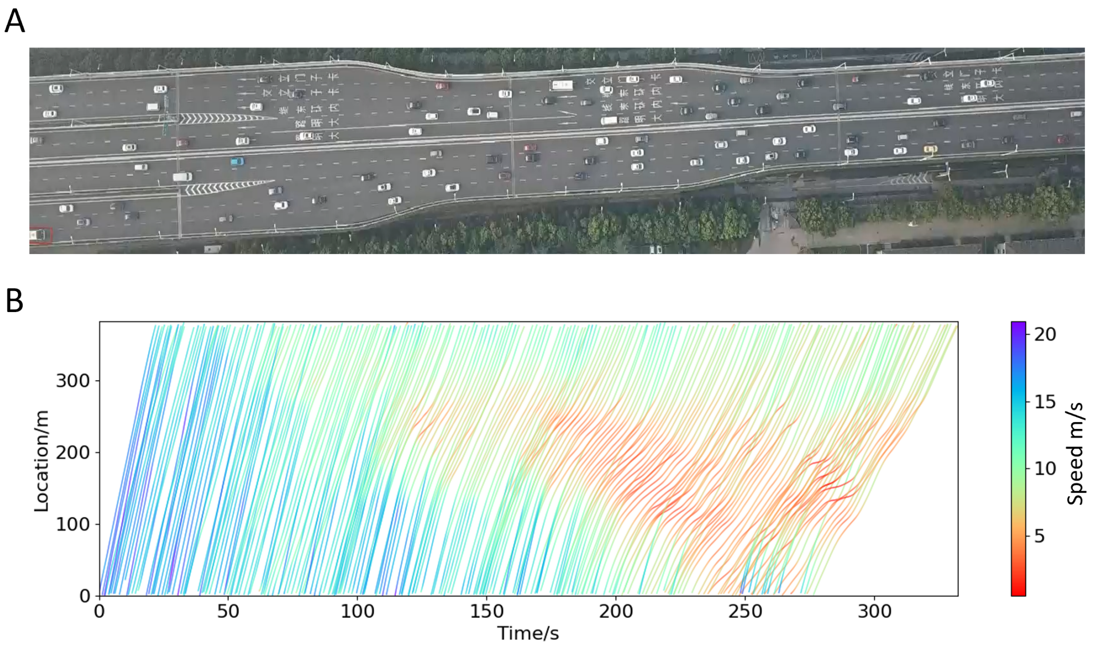

Figure. S1. SQM1 naturalistic driving trajectory dataset. (A) Aerial view of the study site. (B) Corresponding trajectory map.

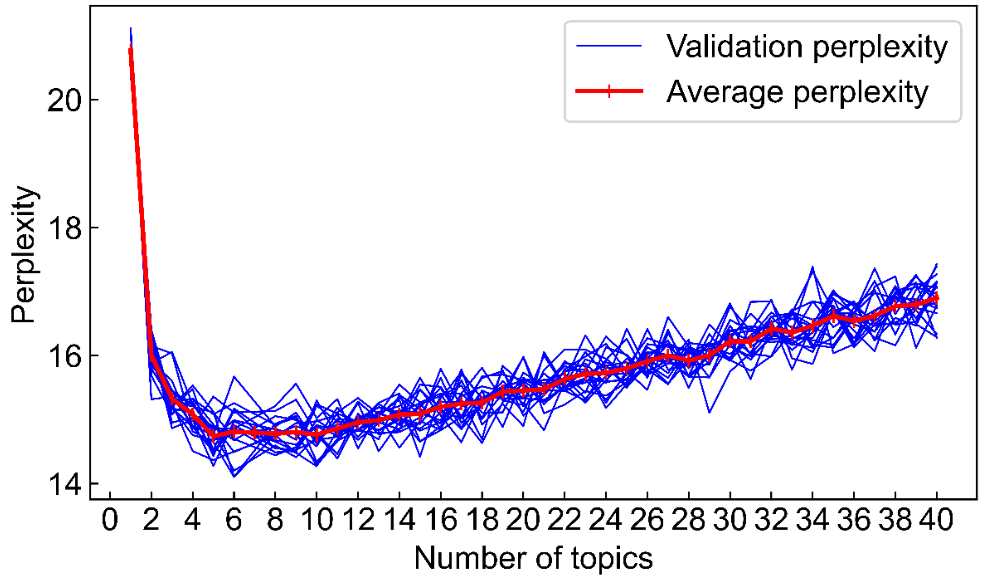

Figure. S2. Perplexity of LDA model with respect to the number of topics. The blue lines represent the perplexity values with respect to the number of topics obtained by repeating the LDA topic estimation 20 times. The red line represents the average perplexity. The optimal topic number was tuned to be five, corresponding to the lowest average perplexity.

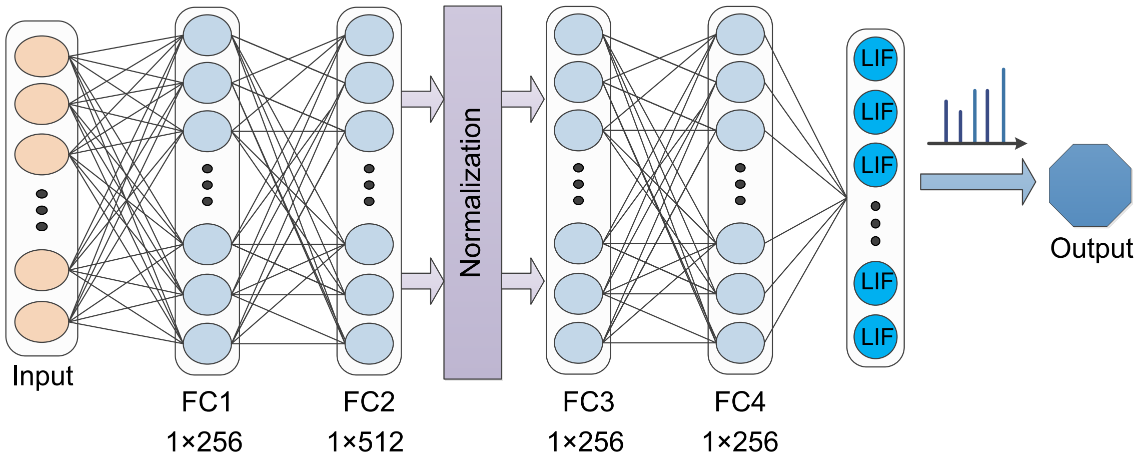

Figure. S3. Network structure of SNN. FC denotes a fully connected layer. The number of neurons in each layer is 256, 512, 256, and 256, respectively. The layer between FC2 and FC3 is a normalization layer for normalizing the output of the FC2 layer. LIF denotes leaky integrate-and-fire neuron, which turns continuous feature to discrete neural spiking.

Supplementary Tables

Table S1. Comparison of theta-band (4-8Hz) EEG power in temporal lobes during driving and resting tasks

Frequency (Hz) ($n = 18$)		Driving ($\bar{x} \pm \hat{\sigma}$, dB)	Resting ($\bar{x} \pm \hat{\sigma}$, dB)	p-value
Left temporal lobe	4	10.53±1.49	9.45±1.35	0.022*
	5	8.66±1.52	7.59±1.43	0.021*
	6	7.23±1.04	6.25±1.46	0.017*
	7	6.29±1.06	5.08±1.45	0.023*
	8	5.44±0.78	4.31±1.47	0.027*
Right temporal lobe	4	11.44±1.05	10.39±1.18	0.019*
	5	9.64±1.15	8.56±1.21	0.015*
	6	8.53±1.01	7.49±1.44	0.019*
	7	7.69±0.99	6.41±1.61	0.037*
	8	7.00±0.86	5.99±1.69	0.016*

Note: p-value was computed based on paired one-sided t-tests and was adjusted via FDR multiple testing correction, and p<0.05 was considered significant. *p<0.05, **p<0.01.

Table S2. Comparison of EEG power in the auditory cortex during video-watching tasks

Frequency (Hz) ($n = 18$)		Power ($\bar{x} \pm \hat{\sigma}$, dB)	Neighbor ($\bar{x} \pm \hat{\sigma}$, dB)	p-value
Sequential video	1	24.18±1.07	15.29±0.72	<0.001**
	2	17.39±1.42	10.44±0.52	<0.001**
	4	14.16±1.37	7.81±0.72	<0.001**
Random video	1	14.55±1.39	14.38±1.10	0.210
	2	10.38±1.30	10.27±1.12	0.274
	4	9.82±1.35	8.02±0.99	<0.001**

Note: p-value was computed based on paired one-sided t-tests and was adjusted via FDR multiple testing correction, and p<0.05 was considered significant. *p<0.05, **p<0.01.

Table S3. Hyperparameter setting of LDA

Parameter	Value
Dirichlet topic distribution prior α	0.117
Dirichlet topic word distribution prior β	0.172
Number of topics K	5
Learning decay	0.8
Iterations	1200
Passes	20
Chunksize	2000

Table S4. Hyperparameter setting of SNN

Parameter	Value
Learning rate	0.001
Membrane time constant τ	5
Parameter optimizer	'Adam'
Firing times of the spike	50
Training epoch	200

Table S5. Multi-step common driving topic prediction errors

Prediction step	1 s	2 s	3 s	4 s	5 s	6 s	7 s	8 s	9 s	10 s
Accuracy (%)	98.14	96.93	95.94	95.52	95.33	95.39	95.56	95.60	95.41	95.00
RMSE	0.06	0.08	0.09	0.09	0.09	0.10	0.10	0.10	0.09	0.09

Table S6. Multi-step longitudinal speed prediction errors

Prediction step	Proposed		DNN		MSVR	
	RMSE (m/s)	MAE (m/s)	RMSE (m/s)	MAE (m/s)	RMSE (m/s)	MAE (m/s)
1 s	2.24	1.18	1.10	0.46	1.17	0.37
2 s	2.24	1.17	1.68	0.96	1.74	0.80
3 s	2.28	1.21	2.02	1.29	2.06	1.10
4 s	2.28	1.22	2.32	1.61	2.35	1.40
5 s	2.32	1.23	2.49	1.82	2.61	1.68
6 s	2.39	1.28	2.67	2.03	2.82	1.87
7 s	2.44	1.33	2.84	2.19	3.01	2.04
8 s	2.54	1.43	2.93	2.28	3.11	2.14
9 s	2.60	1.51	3.01	2.38	3.18	2.25
10 s	2.69	1.62	3.04	2.42	3.27	2.36

Table S7. Multi-step lateral speed prediction errors

Prediction step	Proposed		DNN		MSVR	
	RMSE (m/s)	MAE (m/s)	RMSE (m/s)	MAE (m/s)	RMSE (m/s)	MAE (m/s)
1 s	0.21	0.14	0.16	0.12	0.16	0.12
2 s	0.21	0.14	0.19	0.14	0.19	0.14
3 s	0.21	0.14	0.19	0.15	0.19	0.15
4 s	0.21	0.14	0.19	0.15	0.19	0.15
5 s	0.21	0.14	0.20	0.15	0.20	0.15
6 s	0.21	0.14	0.20	0.16	0.19	0.15
7 s	0.21	0.14	0.20	0.16	0.20	0.16
8 s	0.21	0.14	0.20	0.16	0.20	0.16
9 s	0.21	0.14	0.20	0.16	0.20	0.15
10 s	0.21	0.14	0.20	0.16	0.19	0.15
